# Community health workers: developing roles in public health dementia efforts in the United States

**DOI:** 10.3389/fpubh.2025.1616404

**Published:** 2025-07-02

**Authors:** Elma Johnson, Mickal Lewis, Alexandra Nordyke, Matthew Lee, Shelby Roberts, Joseph E. Gaugler, Soo Borson

**Affiliations:** ^1^School of Public Health, University of Minnesota-Twin Cities, Minneapolis, MN, United States; ^2^BOLD Public Health Center of Excellence on Dementia Caregiving, School of Public Health, University of Minnesota-Twin Cities, Minneapolis, MN, United States; ^3^Alzheimer’s Association, Chicago, IL, United States; ^4^BOLD Public Health Center of Excellence on Dementia Risk Reduction, Alzheimer’s Association, Chicago, IL, United States; ^5^Division of Geriatric Medicine and Palliative Care, NYU Grossman School of Medicine, New York, NY, United States; ^6^BOLD Public Health Center of Excellence on Early Detection of Dementia, NYU Grossman School of Medicine, New York, NY, United States; ^7^Division of Health and Behavior, NYU Grossman School of Medicine, New York, NY, United States; ^8^Keck School of Medicine, University of Southern California, Los Angeles, CA, United States

**Keywords:** chronic disease, community health workers, dementia, public health, workforce, social determinants of health

## Abstract

Community Health Workers (CHWs) are a growing part of the healthcare workforce. Trusted in their communities, CHWs can provide essential health education and connection with culturally responsive health and support resources and programs. Despite their demonstrated effectiveness in improving outcomes in other chronic diseases such as diabetes, hypertension, HIV, and pediatric asthma, CHWs have been underutilized in dementia-related efforts. Properly equipped with education and skills, CHWs can fill important gaps throughout the dementia care continuum, strengthening public health efforts to support people with dementia and their families, especially in populations at higher risk such as African American, Latino and American Indian/Alaska Native groups. We outline key roles CHWs can play throughout the continuum of dementia care, improving brain health and reducing dementia risk at all life stages, such as improving early detection of cognitive impairment and helping caregivers navigate the daily challenges of dementia care in the community setting. Finally, we highlight key actions public health can lead to support the development of a dementia-capable workforce nationwide.

## 1 Introduction

In the United States (U.S.), 129 million people over the age of 18 have at least one chronic condition, and chronic diseases account for 90% of all health expenditures in the U.S (1). Environmental and socioeconomic conditions, such as the built environment, healthy food, and employment status influence health and risk of disease more than genetic factors or access to healthcare services (2). Recognizing the need to address nonmedical influences on health and consider non-traditional ways of delivering care, the U.S. healthcare and public health systems have embraced a more holistic and collaborative approach, integrating medical with community-based social care to promote community-clinical linkages. Supporting cross-sector coordination among healthcare systems, social services, and community organizations, this evolving concept of care recognizes that each sector has a role in addressing social determinants of health and improving the community’s health. This holistic view of health is also at the core of the World Health Organization’s growing Age-Friendly Cities and Communities movement. Age-friendly communities are environments designed to support the autonomy, inclusion and contribution of older adults (3). A related U.S. initiative, Dementia Friendly America, focuses specifically on promoting inclusion and quality of life for people with dementia and their care partners (4). Both age-and dementia-friendly community movements emphasize the social dimensions of health and rely on a variety of community partners and care roles to facilitate coordination of health and social care across settings for older adults and their care partners (5).

Among chronic diseases, dementia presents a compelling case for integrated care. Dementia, a broad category of brain disorders that affect cognitive function, causes difficulties with memory, thinking, and other skills that interfere with daily life and the person’s ability to interact with others (6). Dementia is a significant cause of disability and dependence. These effects extend beyond the individual to families that provide care and the communities where individuals and families live. The needs of people living with dementia call for a flexible social support network able to assist not only immediate family but also friends, community members, and organized service providers. Research has established that lower educational attainment and many forms of social disadvantage are associated with a higher likelihood of dementia and are concentrated in ethnically-and racially-minoritized groups, including African American, Hispanic, American Indian, and some Asian American subgroups. Gaps in the dementia care continuum disproportionately affect these groups and are patterned by social, educational, and economic disadvantage (7). Dementia also carries substantial societal financial costs, especially in publicly funded health and social care. In 2024 alone, the cost of all healthcare and long-term care for people with dementia was estimated to be $360 billion (6). These monetary costs are just one aspect of the total cost of dementia; unpaid family caregivers in the community provide over 80% of all dementia care (8). Finally, dementia is a complex, long-term chronic condition that affects the co-morbidity and management of other chronic conditions. As the person loses the capacity to make independent decisions and direct their own care, managing co-occurring chronic conditions such as heart disease, diabetes, depression, and frailty can become more challenging (9) and requires changes in the roles and interactions of clinicians and family members. As many as 90% of people with dementia also have at least one other chronic disease (7), and the majority have several. Caring for someone with dementia ultimately entails managing functional, behavioral, medical, financial, social, and legal aspects of the person’s life, and requires extensive coordination between clinicians, others on the healthcare team, community services, and family.

It is now widely understood that our healthcare, public health, and social systems are not well prepared to respond to the extent and complexities of dementia care management. The size of our professional dementia-capable healthcare and social services workforce is insufficient to meet current and future demand for dementia care (for an analysis of this topic, see The Alzheimer’s Association, *2024 Alzheimer’s Disease Facts and Figures* report). Addressing the many social and economic health needs of people with dementia falls outside of the expertise and boundaries of traditional healthcare, often leaving family caregivers to take on full responsibility for coordinating services (9). The realities of dementia care call for developing a more diverse array of healthcare workers to bridge dementia care across settings. At this intersection of healthcare delivery, community care, and public health, Community Health Workers (CHW) emerge as one part of the solution that can align and advance the goals of all sectors. The goal of this paper is to illustrate the potential roles of CHWs in dementia risk reduction, detection and care, and how nationally recognized CHW core competencies can be applied to help fill gaps in the dementia care continuum.

## 2 Community health workers: a key part of the public health workforce

The American Public Health Association (10) defines Community Health Workers (CHWs) as *“frontline public health workers who are trusted members of and/or have an unusually close understanding of the community served. This trusting relationship enables CHWs to serve as a liaison/link/intermediary between health/social services and the community to facilitate access to services and improve the quality and cultural competence of service delivery. CHWs also build individual and community capacity by increasing health knowledge and self-sufficiency through a range of activities such as outreach, community education, informal counseling, social support and advocac*y.”

Data from the U.S. Bureau of Labor Statistics identifies 67,200 CHWs in the U.S. in 2022, and the Bureau projects this number to grow by about 14% by 2032 (11). CHWs may hold many job titles within their employing organizations, including peer navigators, lay health workers, community health advocates, community health representatives, peer educators, promotor de salud, and outreach specialists (12–14), and can perform various essential and complementary functions.

Recognizing the potential contribution of CHWs to addressing healthcare delivery shortfalls, various efforts are underway to define professional standards and competencies for the growing CHW workforce and integrate them into healthcare. Within the CHW profession, the National Council on CHW Core Consensus (C3) Standards, formerly the C3 Project, is a national initiative that aims to provide cohesion to the field and increase visibility and understanding of the full potential of CHWs. The National C3 Council has developed a single set of core roles and competencies for CHWs, regardless of their work setting (Table 1) (15). Together, the core roles and competencies define the scope of work, skills, and qualities for CHWs, and serve as a foundational framework to develop training and improve policies to strengthen and expand the CHW workforce.

**Table 1:** Core roles and competencies of community health workers^a^.

10 core roles	11 core competencies
1. Cultural mediation among individuals, communities, and health and social service systems	1. Communication skills
2. Providing culturally appropriate health education and information	2. Interpersonal and relationship-building skills
3. Care coordination, case management, and system navigation	3. Service coordination and navigation skills
4. Providing coaching and social support	4. Capacity building skills
5. Advocating for individuals and communities	5. Advocacy skills
6. Building individual and community capacity	6. Education and facilitation skills
7. Providing direct service	7. Individual and community assessment skills
8. Implementing individual and community assessments	8. Outreach skills
9. Conducting outreach	9. Professional skills and conduct
10. Participating in evaluation and research	10. Evaluation and research skills
	11. Knowledge base

a Source: The National Council on CHW Core Consensus (12).

At the state level, there has been increased action to standardize and formalize the CHW workforce by implementing certification programs for CHWs (16) and identifying stable funding sources to sustain the CHW workforce (17). At the federal level, the U.S. Department of Health and Human Services launched the Community Health Worker Training Program in 2022, which aimed to increase the number of CHWs and support training and apprenticeship programs nationwide (18).

Many chronic disease interventions have incorporated CHWs because of their potential to improve chronic disease outcomes in low-income, underserved, and minoritized communities (19). CHWs have been essential in rural areas providing health services, education, and support where other services and resources may be limited (20). CHWs have also been involved in dementia programming, although to a lesser extent than in other chronic conditions. Promising programs exist in California (21), Minnesota (22) and Missouri (23), among others. However, they remain localized success stories, and their learnings have not broadly informed recommendations for dementia-specific training and development initiatives for CHWs. Additionally, without a coordinated effort to inventory CHW-led programs, the full scope and extent of CHWs’ work in dementia-related programming is unknown, and research on their contributions to dementia services and care is still limited (24).

Given CHWs’ success in managing and improving outcomes for other chronic conditions, including cardiovascular disease (25), diabetes (26), and HIV (27), CHWs, if properly prepared, can effectively address dementia. CHWs can increase awareness of dementia risk factors, facilitate care transitions from community to primary to specialty care, and help caregivers and people with dementia find and access community resources (24). Thanks to their work in the healthcare, community, and social service settings and their deep knowledge of and trusted role within the community, CHWs are ideally positioned to support people with dementia and their caregivers throughout the continuum of dementia care.

## 3 National momentum to transform dementia care: opportunities to engage CHWs

Two important national-level initiatives that aim to transform dementia care are underway: a healthcare-centered initiative to link medical and social care, and the creation of a national public health infrastructure dedicated to brain health and cognitive decline and dementia. CHWs can play vital roles in both.

In the healthcare sector, the Centers for Medicare and Medicaid Services (CMS) Innovation is testing a new payment mechanism for comprehensive dementia care, initiated in summer 2024. The Guiding an Improved Dementia Experience (GUIDE) Model (28) integrates social care into the delivery of healthcare and provides support for both people with dementia (care planning and coordination) and their caregivers (connections to evidence-based education, training, and community-based services). GUIDE’s innovations are two-pronged: it creates a dementia-specific alternative payment model and defines a new role—that of *care navigator* (29). Care navigators are specifically trained for the role of coordinating care and support services for people with dementia and their caregivers across health and social care settings, a role that is well suited for CHWs.

In the U. S., the 2018 Building Our Largest Dementia Infrastructure for Alzheimer’s Act (BOLD Act) (Public Law 155–406) consolidated more than a decade of dementia advocacy efforts after Congress first appropriated public health funding for Alzheimer’s Disease in 2005 (30). The BOLD Act empowers the Centers for Disease Control to build a national public health infrastructure to promote dementia risk reduction, improve early detection and diagnosis of dementia, and support dementia caregivers. BOLD supports three national Public Health Centers of Excellence, each Center addressing one of these three dimensions of public health prevention; currently, 43 state, local, territorial, and tribal health organizations also receive BOLD funding to support their own public health efforts. Implementation of the BOLD Act is driven nationally by the Healthy Brain Initiative Road Map for State and Local Public Health (Road Map) (31). The Road Map provides direction and suggests actions for state and local health departments to improve brain health in their communities. An important domain of public health action the Road Map prioritizes is the development of a *diverse and skilled workforce*, recognizing the urgent need to diversify and grow the current healthcare and social services workforce to support the growing population affected by dementia.

CHWs can play fundamental roles in assuring the success of both BOLD and the GUIDE Model. When positioned at the intersection of public health, clinical care, and community services, CHWs and other direct care workers can connect all three sectors in service to individuals and care partners and augment each sector’s workforce capacity. Properly trained in the subject matter of dementia, dementia care, and social needs, they could extend to dementia their long-valued roles in other areas of public and community health practice.

## 4 Roles of CHW across the dementia care continuum

CHWs’ work is applicable throughout the continuum of dementia care and across settings where they may work to help care for people with dementia. Table 2 provides examples of dementia-related activities that CHWs can do to support people with dementia and their families throughout the disease continuum, aligned with the 10 Core Professional CHW Roles.

**Table 2:** CHW roles across the continuum of dementia care.

Dementia risk reduction	Early detection of dementia	Dementia care and caregiver support
CHW core role: cultural mediation among individuals, communities, and health and social service systems		
Work with communities and health systems to understand what culturally-and linguistically appropriate resources about brain health and healthy brain habits are needed, and collaborate with community partners to adapt existing materials, as needed, for different communities.	Review and help tailor resources to educate culturally and linguistically diverse communities about: Detection and diagnosis processes, including why, how, and where to obtain cognitive assessments. How to navigate healthcare and social services systems after receiving a dementia diagnosis.	Educate communities about what healthcare providers, community organizations, and social services can do to support individuals with dementia and those around them. Educate healthcare and social service providers about cultural practices, values, community beliefs, and priorities surrounding dementia and caregiving.
CHW core role: provide culturally appropriate health education and information		
Integrate culturally centered brain health information and messaging into appropriate health and chronic disease education programs across the life course. Include brain health in community events by organizing or participating in health fairs about healthy lifestyles and chronic disease prevention.	Use culturally relevant and culturally tailored approaches to teach communities about: Detecting signals of possible dementia (“warning signs”) Reducing stigma about dementia, cognitive impairment, and memory loss. Conduct community information sessions to raise awareness of why early detection matters. Coach healthcare staff on how to mitigate dementia-related stigma and engage diverse patients in early detection.	Educate caregivers about the impact of caregiving on their health, how to recognize and respond constructively to stress, mental health, burnout, and when to request help. Become familiar with beliefs and attitudes regarding dementia and caregiving in different cultural communities. Activate individuals and communities to ask questions about dementia care from healthcare providers, advocate for the person with dementia, and manage disagreements about care.
CHW core role: care coordination, case management, and system navigation		
Connect community members with health and social services available to address the social determinants of health related to dementia risk (e.g., access to healthy food, education, and work development programs). Refer community members to local Chronic Disease Prevention Programs (e.g., diabetes, heart, blood pressure, etc.) to address these chronic diseases that impact dementia risk.	Refer families to a clinician for complete assessment and diagnosis. Facilitate patient-provider interactions, including assistance with scheduling, transportation, and other support services to help people attend a screening, doctor’s appointments, and other follow-up. Help people living with dementia and their caregivers and families establish care with a primary care provider and follow up on referrals to specialists.	Facilitate conversations about care coordination with family and other care partners, and help caregivers initiate advanced care planning (medical, financial, and legal) conversations. Help caregivers follow up on referrals to medical and ancillary specialists. Encourage caregivers to assess the need for a higher level of care with the primary care provider and provide families with information about options of residential care (e.g., assisted living facilities, supportive housing, skilled nursing homes, etc.) Support caregivers in communicating with the health care team to update the care plan as needed, including discussing decisions that need to be made if symptoms worsen (e.g., end-of-life care).
CHW core role: provide coaching and social support		
Coach and encourage individuals to talk to their primary care provider about brain health and encourage participation in social service and chronic disease prevention programs.	Encourage individuals and families to address memory and cognitive concerns with their primary care provider and get referred to a specialist for diagnosis, if needed	Educate communities about caregiver self-care and well-being and address cultural beliefs that may prevent caregivers from seeking support. Educate caregivers about the importance of maintaining their health, ways to prevent chronic disease, and encourage them to regularly follow up with healthcare providers for their health needs. Connect caregivers with other caregivers in the community for support.
CHW core role: advocate for individuals and communities		
Elevate brain health as a priority for overall community health and well-being. Advocate for including brain health information in social and chronic disease prevention programs. Advocate for the inclusion of questions related to brain health in community assessments.	Support the development of locally tailored and culturally relevant educational campaigns about early detection, incorporating the needs and perspectives of diverse communities. Connect with local community leaders and policymakers to advocate for early detection and improved access to diagnosis in under-resourced communities. Maintain an ongoing presence in the community as a trusted resource for individuals with emerging cognitive difficulties.	Help communities identify primary caregiver(s) and their support networks. Help community organizations apply for funding for culturally responsive caregiver support programs.
CHW core role: build individual and community capacity		
Support other CHWs in integrating brain health and risk reduction into their work. Share training and resource examples with colleagues in national and state CHW groups and public health conferences.	Educate other CHWs and staff on the early signs of dementia and cognitive impairment. Disseminate information about the benefits of early detection and receiving a dementia diagnosis to CHWs and other colleagues.	Provide dementia care training and education to caregivers and help them identify strategies to maximize the independence of the person with dementia, adapt daily routines, and create structure. Work with communities to identify and advocate for accessible support services.
CHW core role: provide direct service		
Screen individuals for high blood pressure, diabetes, and other conditions that could increase the risk for dementia. Share specific actions that can be taken to reduce the risk of cardiovascular disease, diabetes, and obesity. Provide services or resources to encourage behaviors to support brain health (e.g., smoking cessation, helmet use, and opportunities for physical activity.)	Detect signals of possible cognitive impairment by conducting home environmental assessments and addressing concerns and questions from family and community members. Provide social and emotional support to those going through the detection and diagnosis journey.	Help caregivers apply for funding to make dementia-friendly home modifications. Assist caregivers in applying for financial compensation. Connect caregivers and families to linguistically and culturally responsive support and services in the community.
CHW core role: implement individual and community assessments		
Screen households for social needs related to dementia risk, including food security, home/neighborhood environmental assessments, economic stability, and provide referrals to community services. Highlight existing community assessment questions that are related to brain health.	Assess unmet needs and advocate for the use of the Medicare Annual Wellness Visit. Assess barriers within the community to receiving an early diagnosis and develop resources and information to address them.	Screen caregivers and families for unmet health, social, and economic needs. Identify and address risks and signs of elder abuse, exploitation, and neglect. Inventory existing programs, education, and services for caregivers in the community, and identify gaps.
CHW core role: conduct outreach		
Present information at health fairs, farmers’ markets, schools, and other community events about modifiable risk factors for dementia and ways to address them. Attend existing community events or places people gather (e.g., faith communities) to understand needs and perceptions related to brain health and dementia risk reduction.	Partner with academic centers, health departments, community-based organizations, and health systems to present the benefits of early detection (e.g., health fairs) and the importance of tailoring resources for culturally and linguistically diverse communities to address gaps in dementia detection.	Identify and share new linguistically and culturally responsive resources with caregivers and families. Present to local service organizations about caregivers’ needs and programming to support them. Inform healthcare providers about community programs and services for caregivers and people with dementia.
CHW core role: participate in evaluation and research		
Work with academic centers, health departments, community-based organizations, and health systems conducting research to identify community needs and implement and evaluate programs related to dementia risk reduction in their community.	Help conduct community-level surveys about attitudes and perceptions of the importance of early detection and diagnosis. Help conduct local needs and assets assessments to understand facilitators and barriers to early detection and diagnosis.	Assess accessibility and appropriateness of local caregiving resources and materials offered by academic centers, health departments, community-based organizations, and health systems. Document and report major barriers and facilitators for caregiver enrollment in local programs.

## 5 Integrating CHWs in dementia efforts: opportunities for public health leadership

### 5.1 Adopt a life-course approach to brain health and dementia in CHW training

CHWs work with community members in different life stages for programs spanning from maternal and early childhood health to adolescent and young adulthood, to older adulthood, and therefore have opportunities to engage multi-generational households to improve brain health and quality of life for people of all ages and at all stages of dementia. Prior to dementia diagnosis, CHWs can promote brain health and address dementia risk factors. To improve access to diagnosis and quality care, CHWs can provide education, connect community members with primary and specialty medical care, and foster collaboration between community and healthcare organizations. After a diagnosis of dementia, CHWs can play a key role in building a support network for people with dementia and family caregivers within the community. In addition, integrating dementia education into CHW training for other chronic disease efforts can further support the life-course approach to brain health. Because CHWs can work with people within their home and community settings, they can bring a uniquely grounded insight into the challenges individuals face in preventing and managing dementia-related issues and other chronic conditions. They can also provide individualized support and interventions tailored to their unique circumstances, needs, and strengths. Integrating education about brain health, dementia, and the opportunities for intervention across the life course into training and education curricula for CHWs can better prepare them to support overall health, advance equity in brain health, and improve the quality of life for people with dementia and caregivers of all ages. In the Annual Workforce Survey conducted by the National Association of CHWs in 2021, respondents rated “additional CHW training, including continuing education and specialties,” as the most valued influence on their career growth (32).

Many state and local governments are recognizing the potential for CHWs to work in new areas of health and care (17). Content about dementia and caregiving is now being integrated into many education and training programs for CHWs, including a comprehensive program developed in Oklahoma (33). Public health should inventory such promising training curricula, identify strengths and gaps of each, and recommend a set of competencies and scope of practice related to dementia for CHWs nationally. A standard dementia-specific training curriculum aligned with CHW professional roles to support the activities and roles outlined in Table 2 above would establish dementia as a chronic disease for CHW focus and solidify CHWs’ role in broader public health efforts to address dementia. Recommendations for a national dementia-specific curriculum are currently under development by a collaborative group of public health and professional organizations working with CHWs in the U.S. The curriculum will have a core set of dementia-related content and will be flexibly designed to allow for tailoring to the requirements and regulations of local CHW ecosystems (e.g., at the state and organizational levels). Modifications could include different modes of delivery (e.g., virtual, in-person, hybrid), adaptations for varying levels of professional experience of CHWs (e.g., training needs of experienced CHWs who are new to dementia work as opposed to those who are entirely new to working as a CHW), the setting in which CHWs are employed (e.g., hospital-based, CBO-based, health department-based), among others.

### 5.2 Support CHW certification

States and other entities are actively developing certification standards for CHWs to encourage sustained employment and garner support from policymakers. As of March 2024, 23 states have certification programs for CHWs (16). Stable funding sources for the CHW workforce are essential. Medicaid (34) and, more recently, Medicare (35) will reimburse CHW services if recognized by the state where they work. However, CHW certification is state-dependent and not available everywhere. Public health could provide policy leadership and guidance to states around CHW certification so that all certified CHWs are reimbursed in the states where they live and work. Tiered systems for CHW certification that recognize focused education and experience, like the one in South Carolina, can serve as a model for other states looking to create advancement pathways and reimbursement options for CHWs (36).

Public health can further facilitate this process by clarifying the terminology used to describe the CHW role and how it differs from and complements other comprehensive care coordination and medical case management roles (e.g., Care Coordinator, Care Navigator, Social Worker, Dementia Care Specialist, etc.). Finally, public health can encourage dialogue and consensus-building among professional CHW associations and interest groups across states towards a national set of competencies and certification standards adaptable to different state regulations and legislative ecosystems.

In addition to advancements in certification, related structural changes are needed to support and sustain a dementia-capable CHW workforce. Here, public health can help develop safety protocols to protect the physical, mental, and emotional well-being of CHWs working in the field and establish transparent patient-focused standards (e.g., receiving clinical supervision, managing caseloads to account for catchment area and case complexity, navigating complex cases, etc.).

## 6 Conclusion

The need for dementia care is on track to outpace the capacity of the available professional dementia workforce and family caregivers in the coming decades (6). New models of dementia care are emerging from programs like GUIDE, but these programs are pilots, not available to everyone and may focus on the later stages of disease management. CHWs should become an essential part of a national infrastructure for comprehensive dementia care, by both expanding the capacity of a dementia-capable workforce across the life course and facilitating access to comprehensive dementia care for millions of people with dementia and their caregivers. As they do in other chronic diseases, CHWs can play essential roles throughout the continuum of dementia care, increasing education and awareness about risk reduction strategies, supporting early detection in the community setting, and facilitating care transitions and continued support between healthcare, community, and home settings. With their reach across sectors and within communities, CHWs can support the development of both age-friendly and dementia friendly communities in the U.S.

Supporting the development of dementia-specific training for CHWs and creating consistency among state certifications are two important ways public health can strengthen this critical segment of the public health workforce. By strengthening today’s dementia-capable CHW workforce, we can build the collective capacity to improve brain health and dementia care for all.

## Data Availability

The original contributions presented in the study are included in the article/supplementary material. Further inquiries can be directed to the corresponding author.
